# Antimicrobial Resistance of *Salmonella enterica* Isolates from Tonsil and Jejunum with Lymph Node Tissues of Slaughtered Swine in Metro Manila, Philippines

**DOI:** 10.1155/2014/364265

**Published:** 2014-03-04

**Authors:** Kamela Charmaine S. Ng, Windell L. Rivera

**Affiliations:** ^1^Institute of Biology, College of Science, University of the Philippines, Diliman, Quezon City 1101, Philippines; ^2^Natural Sciences Research Institute, University of the Philippines, Diliman, Quezon City 1101, Philippines

## Abstract

Due to frequent antibiotic exposure, swine is now recognized as potential risk in disseminating drug-resistant *Salmonella enterica* strains. This study thus subjected 20 randomly selected *S. enterica* isolates from tonsil and jejunum with lymph node (JLN) tissues of swine slaughtered in Metro Manila, Philippines, to VITEK 2 antimicrobial susceptibility testing (AST). The test revealed all 20 isolates had resistance to at least one antimicrobial agent, in which highest occurrence of resistance was to amikacin (100%), cefazolin (100%), cefuroxime (100%), cefuroxime axetil (100%), cefoxitin (100%), and gentamicin (100%), followed by ampicillin (50%), and then by sulfamethoxazole trimethoprim (30%). Three multidrug-resistant (MDR) isolates were detected. The sole *S. enterica* serotype Enteritidis isolate showed resistance to 12 different antibiotics including ceftazidime, ceftriaxone, amikacin, gentamicin, and tigecycline. This study is the first to report worldwide on the novel resistance to tigecycline of MDR *S. enterica* serotype Enteritidis isolated from swine tonsil tissues. This finding poses huge therapeutic challenge since MDR *S. enterica* infections are associated with increased rate of hospitalization or death. Thus, continual regulation of antimicrobial use in food animals and prediction of resistant serotypes are crucial to limit the spread of MDR *S. enterica* isolates among hogs and humans.

## 1. Introduction

*Salmonella *is a rod-shaped, Gram-negative, oxidase negative, nonspore forming, predominantly peritrichous enterobacterium [1]. It has been reported and recognized as one of the leading causes of food borne illness, causing diarrheal diseases and enteric fever that may be complicated by extraintestinal infections, such as bacteremia, meningitis, and osteomyelitis, leading to millions of cases of hospitalizations and deaths worldwide each year [2, 3]. It has been isolated from a wide variety of animals, of which swine are the most commonly recognized carriers [4].

The demand for the production of quality livestock meat is increasing. However, the hog livestock production system, despite being the top livestock industry in the Philippines, is constantly challenged with various microbial diseases such as salmonellosis that lead to morbidity-linked reduction in productivity and increased cost of disease treatment [5]. The threat and prevalence of this disease in the country continue to be high [6]. Food poisoning outbreaks and livestock infection caused by *Salmonella *spp. are widespread in the Philippines as evidenced by cases of food poisoning reported in Benguet, Tondo, Manila, and Bulacan and cases of hog morbidity and mortality in Tacloban and Leyte [6–9].

The widespread use of antibiotics has resulted in the emergence of drug-resistant *Salmonella *strains. Since antibiotics are widely used for growth promotion and disease treatment in commercial swine production systems, swine is now recognized as a potential risk in disseminating multidrug-resistant (MDR) *Salmonella *spp. strains [4, 10, 11]. The VITEK 2 system (bioMerieux) has revolutionized antimicrobial susceptibility testing through its rapid and automated fluorescence-based technology. Livermore and coworkers [12] have commended the accuracy of identification and antimicrobial susceptibility testing (AST) of the VITEK 2 system and the significantly reduced handling time that enhances the work flow of clinical microbiology laboratory.

This study aimed to characterize *S. enterica *isolates from tonsil and jejunum with lymph node (JLN) tissues of swine at slaughter in selected accredited and non-accredited meat establishments in Metro Manila. In order to detect MDR strains and shed light on the appropriate treatment against the pathogen, VITEK 2 AST was performed in this study.

## 2. Materials and Methods

### 2.1. Sample Collection

Tonsils and their corresponding JLN tissues were collected from 30 hogs in each of the four non-accredited meat establishments in Quezon City and four accredited slaughterhouses in Malabon, Makati, Pasig, and Quezon City in Metro Manila, Philippines. A 15 cm long segment of JLN was secured with sterile threads on both ends and excised with the flame sterilized knife of the butcher. It was immediately transferred to a sterile bag that was cooled during transport to the laboratory. Afterwards, 25 g of JLN was weighed in a sterile foil and pre-enriched with 225 mL of buffered peptone water in a sterile bottle, agitated for 2 min, and incubated for 18–24 h at 37°C. The tonsil tissues were collected using flame sterilized forceps and butcher's knife and were pre-enriched the same way as the intestinal samples.

### 2.2. Single-Enrichment Broth Culture Method

One hundred microliters of pre-enriched tonsil tissue and JLN samples were inoculated into Rappaport-Vassiliadis Broth (10 mL) while one mL of the pre-enriched samples was inoculated into Tetrathionate Broth (10 mL) and was incubated at 37°C for 24 h. After incubation, broth cultures were streak-plated onto selective, chromogenic medium, Rainbow Agar *Salmonella *(RAS).

### 2.3. DNA Extraction

Three colonies of *Salmonella* spp. cells from RAS were suspended in 150 *μ*L of sterile distilled water. The suspension was heated at 100°C for 10 min and cooled to room temperature afterwards. The cell debris was pelleted by centrifugation at 13,000 rpm for 2 min. The clear supernatant obtained was used as DNA template in PCR [14]. Concentration of DNA extracts was then measured using NanoDrop 2000 following the manufacturer's instructions.

### 2.4. PCR-Based Identification of *Salmonella* spp. Isolates

*InvA* primers, *invA*-F and *invA*-R, which amplify a 244 bp fragment of the *invA* gene specific for *Salmonella* spp. were used for initial detection and confirmation of suspected *Salmonella* spp. isolates [15]. Promega GoTaq Green Master Mix consisting of GoTaq DNA polymerase, 2X Green GoTaq Reaction Buffer, 3 mM MgCl_2,_ and 0.4 mM dNTPs was used for PCR amplification of *invA* region. DNA amplification was performed in a reaction volume of 25 *μ*L. PCR was performed under the following cycling conditions: an initial denaturation at 95°C for 2 min, followed by 35 cycles of denaturation at 95°C for 30 s, annealing at 56°C for 30 s, and extension at 72°C for 2 min. Final extension was done at 72°C for 5 min. For each run, DNA from *S. enterica* serotype Typhimurium was used as positive control while sterile water as template was included as negative control.

Amplicons were checked by separating PCR products through agarose gel electrophoresis in 1x TAE buffer at 100 volts for 30 to 40 min. All PCR products were analyzed in a 1.5% agarose gel stained with 0.5 *μ*g/mL ethidium bromide for 20 min and visualized on a UV transilluminator. The sizes of the bands were estimated using Vivantis 1000 bp DNA ladder as molecular weight marker.

### 2.5. DNA Sequencing of Selected Amplicons

PCR products obtained with the primers representing each serogroup were sent to Macrogen, Inc. (Seoul, South Korea) for purification and DNA sequencing for validation of their identities. Nucleotide sequence data obtained were checked in BioEdit v. 7.0.9.0 sequence alignment program [16] and compared to available sequences of *Salmonella *spp. in GenBank using the Basic Local Alignment Search Tool (BLAST) algorithm available in the National Center for Biotechnology Information website (http://www.ncbi.nlm.nih.gov/BLAST).

### 2.6. Antimicrobial Susceptibility Testing

VITEK 2 AST of 20 randomly selected *S. enterica* isolates from slaughtered swine in both accredited and non-accredited meat establishments was performed to generate the antibiograms and detect MDR strains (Table 1). The stock culture strains were subcultured onto *Salmonella-Shigella* agar plates to confirm their purity. The turbidity of the bacterial suspensions was adjusted with a densitometer (DENSICHEK) to match that of a McFarland 0.4–0.6 standard in 0.45% sterile sodium chloride solution. The time interval between suspension preparation and card filling was less than 30 min to avoid changes in turbidity. Afterwards, the VITEK 2 AST N091 antimicrobial susceptibility cards and bacterial suspension in tubes, both contained in a cassette, were manually loaded into the VITEK 2 system. Each test card was automatically filled with a bacterial suspension, sealed, incubated, and read by kinetic fluorescence measurement. The reporting time for the direct testing of susceptibility against the 17 antibiotics for 20 swine tissue culture isolates by the VITEK 2 system ranged from 8.5 to 10.5 hours.

## 3. Results

VITEK 2 AST of 20 randomly selected *S. enterica *isolates from slaughtered swine in Metro Manila, Philippines (Table 1) revealed that all had resistance to at least one antimicrobial agent, in which highest occurrence of resistance was to amikacin (100%), cefazolin (100%), cefuroxime (100%), cefuroxime axetil (100%), cefoxitin (100%), and gentamicin (100%), followed by ampicillin (50%), and then by sulfamethoxazole trimethoprim (30%). Tables 2(a) and 2(b) show the complete antibiogram of *S. enterica* isolates generated through VITEK 2 AST while Tables 3 and 4 reflect the distribution of in vitro and in vivo antimicrobial resistance, respectively, of *S. enterica* serotypes detected. As seen in Tables 2(a) and 2(b), four *S. enterica *serotype Typhimurium isolates, the sole serotype Heidelberg, one serotype Choleraesuis, the sole serotype Enteritidis, and three serotype Weltevreden isolates were resistant to ampicillin; and two *S. enterica *serotype Typhimurium isolates, and one each from serotypes Heidelberg, Choleraesuis, Enteritidis, and Weltevreden, were resistant to sulfamethoxazole trimethoprim. In addition, the sole serotype Enteritidis was found to be resistant to ceftriaxone, ertapenem, tigecycline (Table 3), and ceftazidime (Table 4). Out of the 20 randomly selected *S. enterica* isolates, three (15%) MDR serotypes were detected in the study, namely, Choleraesuis and Enteritidis, from non-accredited meat establishments and Weltevreden from an accredited meat establishment (Tables 2(a) and 2(b)). Among the three MDR isolates, *S. enterica* serotype Enteritidis was found to be resistant to 12 antibiotics of various antimicrobial classes including third generation cephalosporins ceftazidime and ceftriaxone, third generation aminoglycosides amikacin and gentamicin, as well as to the glycylcycline tigecycline (Tables 3 and 4). VITEK 2 AST also showed that all 20 *S. enterica* isolates tested were susceptible to ceftazidime, cefepime, imipenem, meropenem, amikacin, and levofloxacin (Tables 2(a) and 2(b)). Moreover, VITEK 2 AST demonstrated that *S. enterica* isolates classified under the same serogroup had varying antibiograms as shown in Tables 2(a) and 2(b) for isolates Lt3 and Mbi8.

## 4. Discussion

AST is traditionally performed through the Kirby-Bauer disc diffusion assay. However, this method is laborious and prone to inconsistencies, subjectivity, and human error. The VITEK 2 system (bioMerieux) has revolutionized AST through its rapid and automated fluorescence-based technology that allows determination of minimum inhibitory concentration (MIC) by the analysis of growth kinetics of bacteria with antibiotics in test cards [12].

Among the antibiotics included in the VITEK 2 Gram negative susceptibility card used in the study, piperacillin/tazobactam was suppressed from analysis while ESBL (extra spectrum *β*-lactamase) was not claimed by the machine since the latter is only considered relevant to *Escherichia* and *Klebsiella* species [13]. However, ESBL should not be anymore excluded from AST of *S. enterica* since an ESBL-producing *S. enterica *serotype Typhi has been isolated in the Philippines [17] and many of the isolates tested were found to be resistant to cephalosporins (Table 2(a)), the subsequent characteristic of possessing the enzyme ESBL.

VITEK 2 MIC interpretation guideline is based on Clinical and Laboratory Standards Institute [13]. Referring to Tables 2(a) and 2(b), MIC values and interpretations of almost all of the isolates to cefazolin, cefuroxime, cefuroxime axetil, cefoxitin, amikacin, and gentamicin, and of *S. enterica *serotype Enteritidis to ceftazidime were edited to resistant by the machine for the reason that these antibiotics may only appear active in vitro against *S. enterica* but are not effective in vivo (clinically) and should not be reported as susceptible. This editing of antibiograms based on inferred mechanisms is in agreement with the National Committee for Clinical Laboratory Standards [12].

This study is first to report ampicillin-resistant serotypes of *S. enterica*, namely, Typhimurium, Heidelberg, Choleraesuis, Enteritidis, and Weltevreden, isolated from tonsil and JLN tissues of slaughtered swine in the Philippines using VITEK 2 AST. The routine administration of ampicillin for gastroenteritis in both man and swine and its common use for sensitivity testing in diagnostic laboratories led to the occurrence of ampicillin-resistant *S. enteric *strains [18].

Amoxicillin/clavulanic acid-resistant serotypes of *S. enterica*, namely, Enteritidis and Weltevreden from tonsil and JLN tissues of slaughtered swine in Metro Manila, Philippines, were also first detected in this study using VITEK 2 AST (Table 2(a)). Resistance to cefazolin, cefuroxime, cefuroxime axetil, and cefoxitin was revealed by VITEK 2 AST in all 20 isolates tested in this study (Table 2(a)). This is the first report on resistance to the aforementioned cephalosporins of *S. enterica* serotypes Agona, Choleraesuis, Enteritidis, Heidelberg, Typhimurium, and Weltevreden obtained from tonsil and JLN tissues of slaughtered swine in the Philippines using VITEK 2 AST (Tables 3 and 4). It is remarkable to note that the said cephalosporins: cefazolin, cefuroxime, cefuroxime axetil, and cefoxitin are not commonly employed as therapeutic agents or growth promoters in livestock in the Philippines [19]. The emergence of resistance to cephalosporins has been attributed to plasmid-mediated resistance to AmpC (CMY-2) *β*-lactamase [20].

The sole *S. enterica *serotype Enteritidis detected in this study exhibited resistance to third generation cephalosporins ceftazidime and ceftriaxone (Table 2(a)). In view of the high rate of resistance to fluoroquinolone ciprofloxacin, third generation cephalosporins such as ceftazidime and ceftriaxone are suggested as drugs of choice in the treatment of invasive nontyphoid *Salmonella *infections. Resistance to these antibiotics has been emerging due to production of various class A ESBLs and class C cephalosporinases in *S. enterica *strains [21]. Lee and coworkers [10] have detected ceftriaxone resistance only in *S. enterica *isolates under serogroups B and C1 from Taiwan. They did not detect ceftriaxone resistance in *S. enterica *isolates from the Philippines. This study is the first to report on in vitro resistance to ceftriaxone (Table 3) and in vivo resistance to ceftazidime (Table 4) of *S. enterica* serotype Enteritidis isolated from tonsil and JLN tissues of slaughtered swine in the Philippines using VITEK 2 AST.

Ampicillin, amoxicillin/clavulanic acid, and third generation cephalosporins are commonly used to treat complex salmonellosis [21]. The augmenting emergence of resistance to these antibiotics worldwide has brought about huge therapeutic challenge to animal and human medicine. Resistance to ertapenem was observed in isolate Lt25 (serogroup D, serotype Enteritidis) (Table 2(b)). According to the study of Livermore and coworkers [22], ertapenem was the most active agent tested against members of the family Enterobacteriaceae including *Salmonella *spp. as compared to imipenem, cefepime, ceftriaxone, and piperacillin-tazobactam. Based on their broth microdilution experiments, *Salmonella *spp. isolates from Europe and Australia were susceptible to ertapenem. In the study of Su and coworkers [23], they detected a ceftriaxone and ciprofloxacin-resistant *S. enterica* serotype Typhimurium strain which developed carbapenem resistance during ertapenem treatment which they attributed to a single gene mutation in the organism. This is the first report on ertapenem resistance of *S. enterica* serotype Enteritidis from tonsil and JLN tissues of slaughtered swine in the Philippines using VITEK 2 AST.

Amikacin and gentamicin are aminoglycosides that bind to the bacterial 30S ribosome and interfere with protein synthesis. These aminoglycosides have broader spectra of activity than streptomycin and kanamycin [24]. In 1992, Arboleda and coworkers [25] isolated a gentamicin-sensitive *Salmonella *spp. from a piglet in Laguna, Philippines, whereas Maluping in 2005 [26] isolated gentamicin-resistant *S. enterica* serotype Choleraesuis also from the same animal in Bulacan, Philippines. The result obtained in the present study reflects the increasing resistance of *S. enterica *to gentamicin as two gentamicin-resistant *S. enterica* serotype Weltevreden isolates were detected for the first time from tonsil and JLN tissues of slaughtered swine in the Philippines using VITEK 2 AST (Table 3). In vivo resistance against amikacin was found in all 20 *S. enterica *isolates from tonsil and JLN tissues of slaughtered swine, specifically in serotypes Agona, Choleraesuis, Enteritidis, Heidelberg, Typhimurium, and Weltevreden for the first time in the Philippines using VITEK 2 AST (Table 4). This finding is novel and needs further study since aminoglycoside phosphotransferase APH(3′)-I detected in *S. enterica *generates resistance only to kanamycin, neomycin, lividomycin, paromomycin, and ribostamycin [24].

The most alarming resistance of multidrug isolates found was to tigecycline, a broad-spectrum derivative of minocycline and a member of the novel class glycylcyclines [27]. It is considered a promising drug for treating complex infections since it has a bacteriostatic mode of action against a broad spectrum of aerobic and anaerobic, atypical Gram-positive and Gram-negative organisms, and even MDR ESBL-expressing Enterobacteriaceae and carbapenem-resistant strains [27–30]. It is said to circumvent efflux and ribosomal protection, the two most frequent genetic mechanisms of tetracycline resistance. It is also unaffected by the presence of coresistance to unrelated antimicrobials, such as *β*-lactams, aminoglycosides, and quinolones [30].

Fritsche and coworkers [31] found that 95.7% of all Enterobacteriaceae strains tested including *Salmonella *spp. were susceptible to tigecycline. In 2010, Hentschke and coworkers [27] isolated tigecycline-resistant *S. enterica *serotype Hadar with MIC of 16 *μ*g/mL from a human patient in Germany. Results obtained in the present study are remarkably in contrast to the findings of Fritsche and coworkers [31] but in agreement with that of Hentschke and coworkers [27] implicating the augmenting emergence of *S. enterica *strains. Referring to Table 2(b), VITEK 2 AST revealed resistance of isolate Lt25 (serogroup D, serotype Enteritidis) to tigecycline. This is the first report in the world on tigecycline-resistant *S. enterica* serotype Enteritidis with MIC of ≥8 *μ*g/mL from animal source using VITEK 2 AST. This finding is an important contribution to the global data bank of MDR tigecycline-resistant *S. enterica *serotypes. Further characterization of this novel tigecycline-resistant *S. enterica *strain should be consequently done as this may reveal genetic basis of resistance and factors involved in it.

Chu and coworkers [32] isolated trimethoprim resistant-*S. enterica* serotype Virchow from human in Taiwan. VITEK 2 AST performed in this study revealed sulfamethoxazole trimethoprim-resistant *S. enterica* serotypes Typhimurium, Choleraesuis, Enteritidis, and Weltevreden isolates (Tables 2(b) and 3). This result is of huge relevance since this antimicrobial is commonly administered to treat salmonellosis in the Philippines. In 2005, Maluping [26] isolated sulfamethoxazole trimethoprim-resistant *S. enterica* serotype Choleraesuis from swine in Bulacan, Philippines. The present study is consistent with the latter as sulfamethoxazole trimethoprim-resistant *S. enterica* serotype Choleraesuis was isolated from tonsil tissues of slaughtered swine in Metro Manila, Philippines. Additionally, this study is the first to report on detection of sulfamethoxazole trimethoprim-resistant *S. enterica* serotypes, namely, Enteritidis, Heidelberg, Typhimurium, and Weltevreden, from tonsil and JLN tissues of slaughtered swine in the Philippines using VITEK 2 AST. Due to increasing resistance of nontyphoid *S. enterica *serotypes to sulfamethoxazole trimethoprim, it is not anymore considered as appropriate treatment against invasive salmonellosis [33].

Multiple drug resistance is defined as resistance to three or more classes of antimicrobials [31]. MDR *Salmonella *spp. isolates have been reported since the 1960s [11, 30, 32, 33]. This could be attributed to the use of high levels of combinations of antibiotics without adequate supervision or veterinary advice that is common in small-scale hog-raising in the country [19]. Detection of MDR *Salmonella *spp. isolates from Philippine hogs thus deserves great attention.

Cephalosporins and fluoroquinolones are recommended for treatment against strains resistant to ampicillin and sulfamethoxazole trimethoprim [33]. Although isolates resistant to cephalosporins have been detected in the present study, 100% susceptibility of all isolates tested to levofloxacin, a third generation fluoroquinolone was noted (Table 2(b)).

## 5. Conclusion

The results obtained from this study confirm the role of swine as reservoir of MDR *S. enterica*. Moreover, this study detected for the first time in the world, MDR tigecycline-resistant *S. enterica* serotype Enteritidis from animal source using VITEK 2 AST that poses huge therapeutic challenge to animal and human medicine. Hence, there is a need for continuing regulation of antimicrobial use and mandatory antimicrobial susceptibility testing in food animals.

## Figures and Tables

**Table 1:** Sequence similarities (%) of isolates and reference *Salmonella enterica* sequences obtained from GenBank.

Isolate	Region	Serogroup	Strain	Accession number	Query length and cover, *E* value	% Maximum identity
Lt16	rfbJ	B	*Salmonella enterica* subsp. *enterica* serotype Typhimurium str. U288	CP003836.1	677, 97%, 0.0	99%

Lt21	rfbJ	B	*Salmonella enterica* subsp. *enterica* serotype Typhimurium str. U288	CP003836.1	671, 98%, 0.0	99%

Lt24	rfbJ	B	*Salmonella enterica* subsp. *enterica* serotype Typhimurium str. U288	CP003836.1	653, 99%, 0.0	99%

Lt30	rfbJ	B	*Salmonella enterica* subsp. *enterica* serotype Typhimurium str. U288	CP003836.1	686, 95%, 0.0	99%

Lai1	rfbJ	B	*Salmonella enterica* subsp. *enterica* serotype Agona str. SL483	CP001138.1	674, 98%, 0.0	99%

Lai27	rfbJ	B	*Salmonella enterica* subsp. *enterica* serotype Typhimurium str. U288	CP003836.1	672, 95%, 0.0	99%

Lat23	rfbJ	B	*Salmonella enterica* subsp. *enterica* serotype Agona str. SL483	CP001138.1	678, 97%, 0.0	99%

Lat27	rfbJ	B	*Salmonella enterica *subsp. *enterica* serotype Agona str. SL483	CP001138.1	665, 97%, 0.0	99%

Lbt30	rfbJ	B	*Salmonella enterica* subsp. *enterica* serotype Typhimurium str. U288	CP003836.1	684, 97%, 0.0	99%

Lct47	rfbJ	B	*Salmonella enterica* subsp. *enterica* serotype Typhimurium str. U288	CP003836.1	674, 93%, 0.0	100%

Nt4	rfbJ	B	*Salmonella enterica* subsp. *enterica* serotype Typhimurium str. U288	CP003836.1	684, 94%, 0.0	99%

Pt26	rfbJ	B	*Salmonella enterica* subsp. *enterica* serotype Heidelberg str. B182	CP001120.1	662, 98%, 0.0	99%

Lat20	wzxC1	C1	*Salmonella enterica* subsp. *enterica* serotype Choleraesuis str. SC-B67	AE017220.1	490, 99%, 0.0	99%

Lbt18	wzxC1	C1	*Salmonella enterica* subsp. *enterica* serotype Choleraesuis str. SC-B67	AE017220.1	487, 99%, 0.0	99%

Lt25	tyv	D	*Salmonella enterica* subsp. *enterica* serotype Enteritidis str. P125109	AM933172.1	619, 99%, 0.0	99%

Li16	wzxE1	E	*Salmonella enterica* subsp. *enterica* serotype Weltevreden str. 2007-60-3289-1	FR775224.1	344, 100%, 2*e* − 176	99%

Lt3	wzxE1	E	*Salmonella enterica* subsp. *enterica* serotype Weltevreden str. 2007-60-3289-1	FR775224.1	357, 69%, 4*e* − 115	98%

Mbi8	wzxE1	E	*Salmonella enterica* subsp. *enterica* serotype Weltevreden str. 2007-60-3289-1	FR775224.1	354, 99%, 3*e* − 171	98%

Mbi25	wzxE1	E	*Salmonella enterica* subsp. *enterica* serotype Weltevreden str. 2007-60-3289-1	FR775224.1	348, 99%, 3*e* − 180	100%

Pt12	wzxE1	E	*Salmonella enterica* subsp. enterica serotype Weltevreden str. 2007-60-3289-1	FR775224.1	253, 96%, 3*e* − 119	99%

**Table:** (a)

Isolate	Serogroup	*S. enterica *serotype	Antimicrobial^a^																	
			AMP		AMC		CZ		CXM		CXM AX		FOX		CAZ		CRO		FEP	
			M	I	M	I	M	I	M	I	M	I	M	I	M	I	M	I	M	I
Lt16	B	Typhimurium	≤2	S	≤2	S	≤4	*R	4	*R	4	*R	≤4	*R	≤1	S	≤1	S	≤1	S
Lt21	B	Typhimurium	≥32	R	≤2	S	≥64	R	≥64	R	≥64	R	≤4	*R	≤1	S	≤1	S	≤1	S
Lt24	B	Typhimurium	≥32	R	16	I	≤4	*R	4	*R	4	*R	≤4	*R	≤1	S	≤1	S	≤1	S
Lt30	B	Typhimurium	≤2	S	≤2	S	≤4	*R	4	*R	4	*R	≤4	*R	≤1	S	≤1	S	≤1	S
Lai1	B	Agona	≤2	S	≤2	S	≤4	*R	4	*R	4	*R	≤4	*R	≤1	S	≤1	S	≤1	S
Lai27	B	Typhimurium	≤2	S	≤2	S	≤4	*R	4	*R	4	*R	≤4	*R	≤1	S	≤1	S	≤1	S
Lat23	B	Agona	≤2	S	≤2	S	≤4	*R	4	*R	4	*R	≤4	*R	≤1	S	≤1	S	≤1	S
Lat27	B	Agona	≤2	S	≤2	S	≤4	*R	4	*R	4	*R	≤4	*R	≤1	S	≤1	S	≤1	S
Lbt30	B	Typhimurium	≤2	S	≤2	S	≤4	*R	≤1	*R	≤1	*R	≤4	*R	≤1	S	≤1	S	≤1	S
Lct47	B	Typhimurium	≥32	R	8	S	≤4	*R	4	*R	4	*R	≤4	*R	≤1	S	≤1	S	≤1	S
Nt4	B	Typhimurium	≥32	R	4	S	≤4	*R	4	*R	4	*R	≤4	*R	≤1	S	≤1	S	≤1	S
Pt26	B	Heidelberg	≥32	R	4	S	≤4	*R	8	*R	8	*R	≤4	*R	≤1	S	≤1	S	≤1	S
Lat20	C1	Choleraesuis	≤2	S	≤2	S	≤4	*R	4	*R	4	*R	≤4	*R	≤1	S	≤1	S	≤1	S
Lbt18	C1	Choleraesuis	≥32	R	16	I	≥64	R	8	*R	8	*R	≥64*	R	≤1	S	≤1	S	≤1	S
Lt25	D	Enteritidis	≥32	R	≥32	R	≥64	R	≥64	R	≥64	R	≥64	R	4*	*R	≥64	R	2	S
Li16	E	Weltevreden	≤2	S	≤2	S	≤4	*R	≤1	*R	≤1	*R	≤4	*R	≤1	S	≤1	S	≤1	S
Lt3	E	Weltevreden	≥32	R	4	S	≤4	*R	4	*R	4	*R	≤4	*R	≤1	S	≤1	S	≤1	S
Mbi8	E	Weltevreden	≥32	R	4	S	≤4	*R	4	*R	4	*R	≤4	*R	≤1	S	≤1	S	≤1	S
Mbi25	E	Weltevreden	≥32	R	≥32	R	≥64	R	16	*R	16	*R	16	*R	≤1	S	≤1	S	≤1	S
Pt12	E	Weltevreden	≤2	S	≤2	S	≤4	*R	4	*R	4	*R	≤4	*R	≤1	S	≤1	S	≤1	S

^a^AMP: ampicillin; AMC: amoxicillin/clavulanic acid; CZ: cefazolin; CXM: cefuroxime; CXM AX: cefuroxime axetil; FOX: cefoxitin; CAZ: ceftazidime; CRO: ceftriaxone; FEP: cefepime; M: minimum inhibitory concentration (MIC); I: interpretation; *modified by machine (Clinical and Laboratory Standards Institute, 2012 [13]).

**Table:** (b)

Isolate	Serogroup	*S. enterica *serotype	Antimicrobial^a^															
			ETP		IPM		MEM		AN		GM		LEV		TGC		SXT	
			M	I	M	I	M	I	M	I	M	I	M	I	M	I	M	I
Lt16	B	Typhimurium	≤0.5	S	≤1	S	≤0.25	S	≤2	*R	≤1	*R	≤0.12	S	≤0.5	S	≤20	S
Lt21	B	Typhimurium	≤0.5	S	4*	S	≤0.25	S	≤2	*R	≤1	*R	≤0.12	S	2	S	≤20	S
Lt24	B	Typhimurium	≤0.5	S	≤1	S	≤0.25	S	≤2	*R	≤1	*R	≤0.12	S	≤0.5	S	≤20	S
Lt30	B	Typhimurium	≤0.5	S	≤1	S	≤0.25	S	≤2	*R	≤1	*R	≤0.12	S	1	S	≤20	S
Lai1	B	Agona	≤0.5	S	≤1	S	≤0.25	S	≤2	*R	≤1	*R	≤0.12	S	≤0.5	S	≤20	S
Lai27	B	Typhimurium	≤0.5	S	≤1	S	≤0.25	S	≤2	*R	≤1	*R	≤0.12	S	1	S	≤20	S
Lat23	B	Agona	≤0.5	S	≤1	S	≤0.25	S	≤2	*R	≤1	*R	≤0.12	S	≤0.5	S	≤20	S
Lat27	B	Agona	≤0.5	S	≤1	S	≤0.25	S	≤2	*R	≤1	*R	≤0.12	S	≤0.5	S	≤20	S
Lbt30	B	Typhimurium	≤0.5	S	≤1	S	≤0.25	S	≤2	*R	≤1	*R	≤0.12	S	4	I	≤20	S
Lct47	B	Typhimurium	≤0.5	S	≤1	S	≤0.25	S	≤2	*R	≤1	*R	1	S	1	S	≥320	R
Nt4	B	Typhimurium	≤0.5	S	≤1	S	≤0.25	S	≤2	*R	≤1	*R	≤0.12	S	2	S	≥320	R
Pt26	B	Heidelberg	≤0.5	S	≤1	S	≤0.25	S	≤2	*R	≤1	*R	≤0.12	S	2	S	≥320	R
Lat20	C1	Choleraesuis	≤0.5	S	≤1	S	≤0.25	S	≤2	*R	≤1	*R	≤0.12	S	1	S	≤20	S
Lbt18	C1	Choleraesuis	≤0.5	S	≤1	S	≤0.25	S	≤2	*R	≤1	*R	≤0.12	S	≤0.5	S	≥320	R
Lt25	D	Enteritidis	≥8	R	4	S	1	S	≤2	*R	≤1	*R	1	S	≥8	R	≥320	R
Li16	E	Weltevreden	≤0.5	S	4*	S	≤0.25	S	≤2	*R	≤1	*R	≤0.12	S	4	I	≤20	S
Lt3	E	Weltevreden	≤0.5	S	≤1	S	≤0.25	S	≤2	*R	≥16	R	≤0.12	S	≤0.5	S	≤20	S
Mbi8	E	Weltevreden	≤0.5	S	≤1	S	≤0.25	S	≤2	*R	≥16	R	≤0.12	S	≤0.5	S	≥320	R
Mbi25	E	Weltevreden	≤0.5	S	≤1	S	≤0.25	S	≤2	*R	≤1	*R	1	S	2	S	≤20	S
Pt12	E	Weltevreden	≤0.5	S	≤1	S	≤0.25	S	≤2	*R	≤1	*R	≤0.12	S	1	S	≤20	S

^a^ETP: ertapenem; IPM: imipenem; MEM: meropenem; AN: amikacin; GM: gentamicin; LEV: levofloxacin; TGC: tigecycline; SXT: trimethoprim/sulfamethoxazole; M: minimum inhibitory concentration (MIC); I: interpretation; *modified by machine (Clinical and Laboratory Standards Institute, 2012 [13]).

**Table 3:** Distribution of in vitro antimicrobial resistance of *Salmonella enterica* serotypes isolated from swine at slaughter in Metro Manila.

Serotype (number tested)	Number of isolates resistant to the antimicrobial agents^a^										
	AMP	AMC	CZ	CXM	CXM AX	FOX	CRO	ETP	GM	TGC	SXT
Choleraesuis (2)	1		1			1					1
Enteritidis (1)	1	1	1	1	1	1	1	1		1	1
Heidelberg (1)	1										1
Typhimurium (8)	4		1	1	1						2
Weltevreden (5)	3	1	1						2		1

^a^AMP: ampicillin; AMC: amoxicillin/clavulanic acid; CZ: cefazolin; CXM: cefuroxime; CXM AX: cefuroxime axetil; FOX: cefoxitin; CRO: ceftriaxone; ETP: ertapenem; GM: gentamicin; TGC: tigecycline; SXT: trimethoprim/sulfamethoxazole (Clinical and Laboratory Standards Institute, 2012 [13]).

**Table 4:** Distribution of in vivo antimicrobial resistance of *Salmonella enterica* serotypes isolated from swine at slaughter in Metro Manila.

Serotype (number tested)	Number of isolates resistant to the antimicrobial agents^a^						
	CZ	CXM	CXM AX	FOX	CAZ	AN	GM
Agona (3)	3	3	3	3		3	3
Choleraesuis (2)	1	2	2	1		2	2
Enteritidis (1)					1	1	1
Heidelberg (1)	1	1	1	1		1	1
Typhimurium (8)	7	7	7	8		8	8
Weltevreden (5)	4	5	5	5		5	3

^a^CZ: cefazolin; CXM: cefuroxime; CXM AX: cefuroxime axetil; FOX: cefoxitin; CAZ: ceftazidime; AN: amikacin; GM: gentamicin (Clinical and Laboratory Standards Institute, 2012 [13]).
